# Dual Effects of Cold Storage and Stored Host Eggs of *Spodoptera frugiperda* (Smith) (Lepidoptera: Noctuidae) on the Reproductive Capacity of *Telenomus remus* Nixon (Hymenoptera: Scelionidae)

**DOI:** 10.3390/insects15040233

**Published:** 2024-03-28

**Authors:** Ranran Qiu, Jun Li, Nicolas Desneux, Liansheng Zang, Xiaofang He, Xin Lü

**Affiliations:** 1 Guangdong Key Laboratory of Animal Conservation and Resource Utilization, Guangdong Public Laboratory of Wild Animal Conservation and Utilization, Institute of Zoology, Guangdong Academy of Sciences, 105 Xingang Road West, Guangzhou 510260, China; 2 College of Plant Protection, South China Agricultural University, Guangzhou 510642, China; 3 Université Côte d’Azur, INRAE, CNRS, UMR ISA, 06000 Nice, France; 4 National Key Laboratory of Green Pesticide, Key Laboratory of Green Pesticide and Agricultural Bioengineering, Ministry of Education, Guizhou University, Guiyang 550025, China

**Keywords:** stored host, shelf life, mass rearing, biological control, parasitoid

## Abstract

**Simple Summary:**

*Spodoptera frugiperda* is an invasive pest that causes serious damage to many economically important cereal crops worldwide. *Telenomus remus* is one of the most important egg parasitoids against *S*. *frugiperda*. Insect storage at certain conditions that temporarily halt development would facilitate effective commercial rearing and transportation, limiting the usual problems of hatching or emergence during shipping and aiding the inundative release of the parasitoids. We hypothesized that when parasitoid parasitizing host eggs are subjected to cold storage, their reproductive capacity would be affected by both the host quality and the cold storage. To confirm this hypothesis, the effects of cold storage on the reproductive capacity of *T. remus* reared on stored/non-stored *S*. *frugiperda* eggs and the hatching rate of *S*. *frugiperda* larvae were investigated. The results indicated that *S. frugiperda* eggs could only be stored at 10 °C for five days to be suitable for rearing *T. remus*. *Telenomus remus* pupae in non-stored *S. frugiperda* eggs were stored at 13 °C for 15 days without affecting the reproductive capacity of the parasitoid. However, the optimum cold storage conditions for *T. remus* parasitizing stored eggs were 7 °C for 5 days in the larval stage. The maximal shelf lives of *T. remus* parasitizing fresh and cold-stored *S. frugiperda* eggs were 15 and 10 days (including the storage duration of host eggs), respectively. Our study revealed that cold storage affected host eggs, thus further affecting the reproductive capacity of the parasitoid. Different storage conditions should be used when mass-rearing egg parasitoids on stored and non-stored eggs to reduce costs and obtain high-quality parasitoids.

**Abstract:**

*Spodoptera frugiperda* is the preferred host of the parasitoid *Telenomus remus*. Cold storage can preserve a sufficient quantity of parasitoids and their hosts in a laboratory colony for mass release. First, the effects of cold storage on the reproductive capacity of *T. remus* reared on non-stored *S*. *frugiperda* eggs and the hatching rate of unparasitized *S. frugiperda* eggs were investigated. Further, the dual effects of cold storage and stored *S. frugiperda* eggs on the reproductive capacity of *T. remus* were studied to determine the optimal storage conditions and the maximal shelf life for both the host and the parasitoid. The emergence rate, the number of adults produced, and the female proportion of *T. remus* were affected by cold storage factors. Pupae stored at 13 °C for 15 days is optimum for *T. remus* reared on non-stored *S. frugiperda* eggs. *Spodoptera frugiperda* eggs could only be stored at 10 °C for five days to be suitable for rearing *T. remus*. The optimum cold storage conditions for *T. remus* parasitizing stored eggs were 7 °C for 5 days in the larval stage. The maximal shelf lives of *T. remus* parasitizing cold-stored *S. frugiperda* eggs were 10 days. Cold storage affected the hatching rate of *S. frugiperda* eggs, thereby further affecting the reproductive capacity of *T. remus*. The findings suggest that different storage conditions should be used when mass-rearing *T. remus* on stored and non-stored eggs. *Telenomus remus* should be reproduced using fresh laid *S*. *frugiperda* eggs for maximum shelf life.

## 1. Introduction

The fall armyworm (FAW), *Spodoptera frugiperda* (J.E. Smith) (Lepidoptera: Noctuidae), is one of the most economically important invasive pests affecting the world’s cereal crops [1]. It is critical to apply ecologically sound and highly efficient pest management strategies to control this key pest [2]. Natural enemies, especially parasitoids, were widely employed after the FAW invaded China [3,4,5,6,7]. *Telenomus remus* Nixon (Hymenoptera: Scelionidae) is one of the most important egg parasitoids of Lepidopteran insects [8,9]. This key parasitoid has already been used in augmentative biological control programs against *Spodoptera* spp. [6,10,11]. *Telenomus remus* parasitized the newly invaded FAW eggs in Chinese corn fields, with FAW egg mass parasitism rates of 30% in the first season [12]. After the mass release of *T. remus*, the parasitism rate reached up to 90%, and the application of insecticides was reduced by 49–80% [11]. Regular release of mass-reared parasitoids is necessary to maintain the population at the required level for pest control once the host population has declined in the field [13,14,15]. A large-scale release of *Trichogramma dendrolimi* has been used successfully against *Ostrinia furnacalis* in northeastern China [16]. Nearly 200,000–400,000 *Aphytis melinus* wasps per acre are periodically released to control the California red scale on citrus crops, and the parasitoid *Muscidifurax raptor* has been employed to control house flies, *Musca domestica* [17]. Therefore, mass rearing and quality control are crucial for the successful utilization of *T. remus*. Rearing factors such as low-temperature storage tolerance and cold resistance should be examined with the aim of prolonging the shelf life and increasing parasitoid numbers for mass release [18,19].

*Telenomus remus* can be reared on its natural host (the FAW, *Spodoptera litura* Fabricius (Lepidoptera: Noctuidae) and *Spodoptera exigua* (Hübner) (Lepidoptera: Noctuidae), as well as on alternate hosts *Agrotis biconica* Kollar (Lepidoptera: Noctuidae) [20] and the rice moth *Corcyra cephalonica* Stainton (Lepidoptera: Pyralidae) [21]. However, our previous experiments and other studies in China indicated that *T. remus* could not be successfully reared on rice moth eggs [22]. Using FAW eggs can avoid the problem of decreasing the control performance on natural hosts caused by rearing parasitic wasps on alternative hosts. Mass rearing of the parasitoid on the target host may create intraspecific selection of parasitoids particularly adapted to this host and even increase parasitism success in the field. In addition, selective and non-selective tests showed that *T. remus* preferred FAW eggs to *S*. *litura* eggs. The quality control of mass reproduction of *T. remus* was studied by using FAW eggs as hosts (unpublished). Based on the results above, further studies are conducted here on the cold storage of *T. remus* and its host, FAW eggs.

Previous studies have investigated the effect of cold storage on the parasitic efficiency of the wasps or have studied the storage conditions for parasitic wasps and hosts prior to parasitism to determine the maximal shelf life of parasitoids [23,24,25,26,27,28]. The eggs of *S*. *litura* and *C*. *cephalonica* can be used to reproduce *T. remus* after cold storage. *Telenomus remus* produced from fresh eggs of different hosts varied in storage conditions and had a shelf life of about 5–21 days. We believe that cold storage has an impact on the host eggs as well as the parasitic wasps that they reproduce. We hypothesized that when parasitic wasps that reproduce with cold-stored eggs are further subjected to cold storage, their reproductive capacity would be affected by both the quality of the host and the cold storage. To confirm this hypothesis, we first investigated the effects of cold storage on the reproductive capacity of *T. remus* reared on fresh-laid fall armyworm eggs. Further, the hatching rate of unparasitized FAW eggs under different cold storage conditions and the reproductive capacity of *T. remus* reared on stored FAW eggs were studied to determine the optimum storage conditions for keeping the host eggs fresh and conducive to the reproduction of the parasitoid. Finally, *T. remus* was reared with cold-stored FAW eggs and further subjected to cold storage to determine the response under the dual stresses of cold storage and stored host eggs to determine the optimal storage conditions and measure the actual shelf life.

## 2. Materials and Methods

### 2.1. Insects

The parasitoid *Telenomus remus* and the eggs of its host, *Spodoptera frugiperda*, were originally collected on 28 April 2019, and 20 September 2020, from corn fields in Huadu and Huizhou of Guangdong province, southern China. To establish the laboratory colony, *S. frugiperda* larvae were fed an artificial diet developed by Li et al. [29], and adults were fed with a 10% honey solution. The newly laid eggs were used within 24 h for the subsequent parasitism and cold storage experiments. In the wild, *S. frugiperda* lays single-layer, double-layer, and multi-layer egg masses. *Telenomus remus* can parasitize not only the single-layer egg masses but also the double-layer and multi-layer egg masses [5,9]. However, most of the egg masses were single-layer in the lab. To maintain the consistency of the quality of the experimental egg masses and reduce the error, the natural single-layer egg masses were used in this study. The *Telenomus remus* stock was reared on the eggs of *S. frugiperda* as the host. The duration from egg to adulthood of *T. remus* was about 10 days at 27 ± 1 °C. The eggs hatch into the first instar after 24 h. Then the larvae develop into prepupae after 2 days. Also, after 2 days of development, the prepupae pupate, and the pupa stage lasts for approximately 5 days. The insects were reared in a climatic incubator (Yamato, Tokyo, Japan) set at 27 ± 1 °C, 75 ± 5% RH, and a 16:8 h (L:D) photoperiod.

### 2.2. Experimental Setup

#### 2.2.1. Parasitic Experiment

Before the parasitic experiment, the parasitized egg masses containing ~100 eggs were prepared first. Based on the number of parasitized eggs and the average 85% emergence rate (from thirty parasitized FAW egg masses) of *T. remus*, the parasitized FAW egg masses, containing approximately 80 wasps of both sexes, were placed separately in a glass tube (3 cm in diameter × 9 cm high). After *T. remus* adults emerged and mated for two hours, a newly laid single-layer FAW egg mass with approximately 80 plump eggs was placed in the tube (i.e., a ratio of *T. remus* to FAW eggs was 1:1) in each treatment. The sex ratio (female to male) of *T. remus* was approximately 8:1 in each replicate. Then, the tubes were placed in climatic incubators that were set at 27 ± 1 °C, 75 ± 5% RH, and a 16:8 h (L:D) photoperiod. After 24 h, the *T. remus* adults were removed.

#### 2.2.2. The Effect of Cold Storage on the Reproductive Capacity of *T. remus* on Fresh (Non-Stored) FAW Eggs (Bioassay 1)

The experimental setup followed that of Lü et al. [26], with three factors and five levels (Table 1). The control group was treated with non-cold storage. The developmental stages were examined under a binocular microscope (Olympus, Tokyo, Japan) [22]. Among them, because the pupal period lasts approximately 5 days at 27 ± 1 °C, the first and third days of the pupal period were chosen as storage stages. After the parasitism experiment in Section 2.2.1, the three parasitized FAW egg masses (three replicates) develop to different stages under different rearing conditions. When the wasp progeny had developed to the stages stipulated in the experimental treatment, the FAW egg masses were transferred to low-temperature incubators (Yamato, Tokyo, Japan) set at different temperatures. The control group was reared in the incubators under the same rearing conditions until *T. remus* adults emerged. After completing the set storage period, the parasitized FAW egg masses were put into incubators set at the same rearing conditions as above until the adult wasps emerged. The emergence rate, number of adults produced, and female proportion of *T. remus* were calculated.

#### 2.2.3. The Effect of Cold Storage on the Hatching Rate of FAW Larvae and Rearing *T. remus* on Stored FAW Eggs (Bioassay 2)

The cold storage experimental design comprised two factors with four levels: temperature (4 ± 1 °C, 7 ± 1 °C, 10 ± 1 °C, and 13 ± 1 °C) and storage duration (3 d, 5 d, 7 d, and 9 d), with the response being the hatching rate of FAW eggs. Ten newly laid and single-layer FAW egg masses (~80 eggs) in similar conditions were used in each storage treatment and the control treatment (non-stored). The egg masses were put into low-temperature incubators, each representing a different storage temperature. After completing the set storage period, the FAW egg masses were put into climatic rearing incubators until the FAW larvae hatched. The hatching rate of FAW larvae was calculated.

To determine the most suitable storage conditions for FAW eggs for rearing *T. remus*, the differences in emergence rate, the number of adults produced, and the proportion of female *T. remus* reared on stored and non-stored FAW eggs (control group) were analyzed. Another 30 egg masses were treated with the same cold storage treatments for rearing *T. remus* in the following experiment. *Telenumus remus* parasitized on non-stored FAW eggs was the control group. Each stored FAW egg mass was placed separately in a glass tube (3 cm in diameter × 9 cm high) for exposure to a single newly emerged *T. remus* female adult that had previously mated. After 24 h, the female wasps were removed, and the parasitized egg masses were put into the climatic incubators set at the rearing conditions as above.

#### 2.2.4. The Effect of Cold Storage on the Reproduction Capacity of *T. remus* Parasitizing Stored FAW Eggs (Bioassay 3)

Based on the results of bioassays 1 and 2, *T. remus* reared on the single-layer FAW eggs that had been stored at 10 °C for five days were subjected to cold storage, using treatments of larva-4 °C-5 d, larva-7 °C-5 d, pupa-10 °C-10 d, and pupa-13 °C-15 d (developmental stage-storage temperature-storage duration). The preparation of materials and the experimental methods followed those of bioassay 1. The experiment was set up with three replicates. The ratio of *T. remus* to FAW eggs was 1:1, and the ratio of female to male wasps was approximately 8:1 in each replicate. After the given storage duration and without further acclimation, the storage treatment groups and the control group with non-stored egg masses were put into climatic incubators as in bioassays 1 and 2 until adult wasp emergence. The emergence rate, number of adults produced, and female proportion of *T. remus* were calculated.

### 2.3. Biological Parameters Assessed

After storage, the number of FAW eggs, FAW hatching larvae, and emergence holes of *T. remus* on the parasitized FAW eggs, *T. remus* adults, and female adults were monitored under a binocular microscope (Olympus, Tokyo, Japan). Considering that the attack of egg parasitic wasps on the host usually results in the partial failure of the development of parasitic wasp offspring and the abortion of host eggs [30], the actual number of parasitized eggs included the number of adult emergence and wizened eggs, which were also recorded in consequence. FAW hatching rate, *T. remus* emergence rate, number of adults produced, and female proportion were calculated as follows:

FAW hatch rate (%) = the number of FAW hatching larvae/total number of FAW eggs × 100

Emergence rate (%) = number of adults/(total number of FAW eggs − wizened eggs) × 100

Female proportion (%) = number of female adults/total number of adults × 100

### 2.4. Statistical Analysis

For bioassay 1, a multi-factor analysis of variance (PROC GLM) was used to evaluate the effect of each cold storage factor (temperature, developmental stage, and storage duration) and their interaction on the emergence rate, the number of adults produced, and the proportion of female *T. remus* parasitizing non-stored FAW eggs. To obtain the optimum storage condition of *T. remus* parasitizing non-stored FAW eggs, a one-way analysis of variance (PROC ANOVA) was used to analyze the differences in emergence rate, number of adults produced, and proportion of female *T. remus* between cold-stored treatment groups and the control group.

For bioassay 2, a multi-factor analysis of variance (PROC GLM) was utilized to assess the effect of each cold storage factor (temperature and storage duration) and their interaction on the hatching rate of FAW larvae and on the emergence rate, the number of adults produced, and the proportion of female *T. remus* from stored FAW eggs. A one-way analysis of variance (PROC ANOVA) was conducted to analyze the differences in the hatching rate of FAW larvae between cold-stored treatments and the control group.

For bioassay 3, the actual shelf life of *T. remus* reared on stored FAW eggs was measured to determine the dual effects of cold storage and stored FAW eggs on the reproductive capacity of *T. remus*. One-way analysis of variance (PROC ANOVA) was used to analyze differences in the emergence rate, the number of adults produced, and the proportion of female *T. remus* between cold-stored treatment groups and the control group.

An arcsine square root transformation was performed on the percentage data, and count data of the number of adults were log_10_-transformed to fit a normal distribution before statistical analysis. When means were not normally distributed even when transformed, a nonparametric Kruskal–Wallis ANOVA was used, and means were separated using a Mann–Whitney U test. Dunnett’s test was used in the analysis of storage treatments vs. control groups. Differences were considered significant at *p <* 0.05 in all experiments. All data were analyzed with SPSS 22.0 software (IBM, Armonk, NY, USA).

## 3. Results

### 3.1. The Effects of Cold Storage on the Reproductive Capacity of T. remus Parasitizing Non-Stored FAW Eggs

The developmental stage, storage duration, storage temperature, and the three-way interaction had significant effects on the emergence rate and the number of adults produced, and thus the effects were not independent (Table S1). The proportion of females was not significantly affected by the developmental stage but was significantly affected by the three-way interaction of cold storage factors. The emergence rate, the number of adults produced, and the proportion of females were significantly affected by the storage duration and storage temperature.

**Figure 1:**
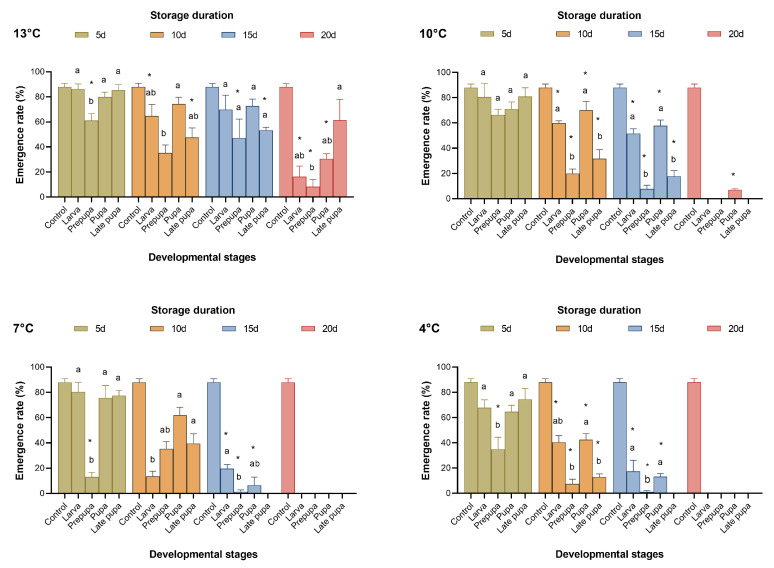
Emergence rate of *Telenomus remus* reared on fresh FAW eggs reared under different cold storage factors vs. controls. Means (± SE) were calculated from three replicates. The asterisks indicate significant differences between storage treatments and control data based on Dunnett’s test at *p* < 0.05. Cold storage treatments with a different lowercase letter under the same storage duration are significantly different using Tukey’s test at *p* < 0.05.

**Figure 2:**
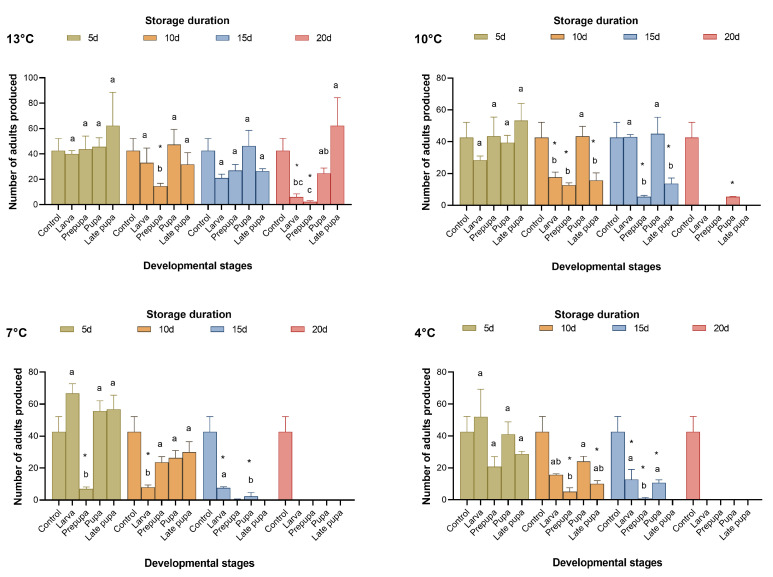
Number of adults produced using fresh FAW eggs for rearing *Telenomus remus* under cold storage treatments vs. controls. Means (± SE) were calculated from three replicates. The asterisks indicate significant differences between storage treatments and controls based on Dunnett’s test at *p* < 0.05. Cold storage treatments with a different lowercase letter under the same storage duration are significantly different using Tukey’s test at *p* < 0.05.

**Figure 3:**
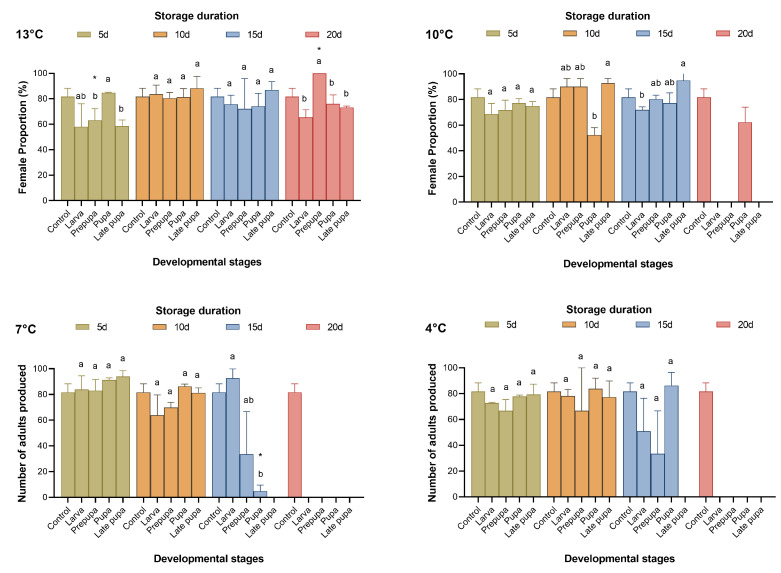
Female proportion of *Telenomus remus* reared on fresh FAW eggs under different cold storage treatments vs. controls. Means (± SE) were calculated from three replicates. The asterisks indicate significant differences between storage treatments and controls based on Dunnett’s test at *p* < 0.05. Cold storage treatments with a different lowercase letter under the same storage duration are significantly different using Tukey’s test at *p* < 0.05.

When *T. remus* was stored at 13 °C, 16.31–61.27% of parasitic wasps emerged after 20 days of cold storage. When the cold storage temperature was below 10 °C, no parasitic wasps emerged after more than 15 days (Figure 1 and Figure 2); as a result, the proportion of females sharply decreased (Figure 3). From the results for the emergence rate and the number of adults produced, the most sensitive developmental stage for cold storage for *T. remus* was the prepupal stage (4 d), especially when the duration of storage was more than five days, where the emergence rate was less than 50%, and fewer than 30 adults were produced (Figure 1 and Figure 2). In contrast, pupal *T. remus* had a stronger tolerance to cold storage, even when the duration of storage exceeded 15 days.

Figure 1, Figure 2 and Figure 3 show that pupae stored at 13 °C for 15 days and at 10 °C for 10 days, as well as larvae stored at 7 °C and 4 °C for five days, had good reproductive performance after cold storage. The emergence rate (*F*_4,10_ = 3.561, 27.732, 18.823, and 18.804; *p* = 0.424, 0.063, 0.299, and 0.294), the number of adults produced (*F*_4,10_ = 2.797, 9.048, 34.578, and 2.022; *p* = 0.999, 0.999, 0.165, and 0.990), and the proportion of females (*F*_4,10_ = 0.231, 4.261, 0.397, and 0.955, *p* = 0.993, 0.175, 0.945, and 0.642) of the four storage conditions were not significantly different from those of the control. Although the performance of late pupa-13 °C-20 d had no significant difference from the control, by 15–20 days of storage, some *T. remus* adults had emerged. Therefore, *T. remus* parasitizing non-stored FAW eggs can be stored for a maximum of 15 days when the pupae are stored at 13 °C; these conditions obtained a 72% emergence rate, 46.33 adults, and a 74.22% female proportion after cold storage.

### 3.2. The Effects of Cold Storage on the Hatching Rate of FAW and T. remus Parasitizing Cold-Stored FAW Eggs

**Figure 4:**
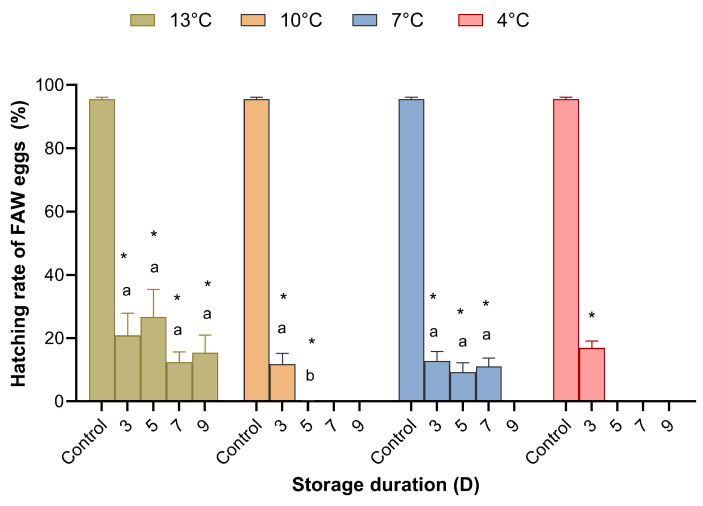
Hatching rate of *Spodoptera frugiperda* eggs stored at different temperatures and for different durations. Means (± SE) were calculated from ten replicates. The asterisks indicate significant differences between storage treatments and control data based on Dunnett’s test at *p* = 0.05. Cold storage treatments with a different lower case letter under the same temperature are significantly different using Tukey’s test at *p* < 0.05.

Temperature and storage duration significantly affected the hatching rate of *S*. *frugiperda* (Table S2), resulting in a less than 30% larval hatching rate, and the difference between treatment and control groups was significant. There were very few FAW larvae produced when the eggs were stored at 10 °C for 5–9 days, 7 °C for 9 days, or 4 °C for 5–9 days (Figure 4).

Using the stored FAW eggs to rear *T. remus*, a multifactor analysis of variance revealed that the temperature and storage duration affected the host eggs and hence the reproductive capacity of the parasitoids (Table S3). The emergence rate, the number of adults produced, and the proportion of female parasitoids gradually decreased as the host eggs were kept at lower temperatures and for longer periods. However, the host eggs being stored at 13 °C did not have an optimal effect on the reproduction of *T. remus.* The FAW eggs stored at 10 °C had an optimal effect on the reproduction of *T. remus* under the same duration of storage (Table 2). *T. remus* obtained the best results, with emergence rates of 82.87% and 76.05%, 26.30, and 27.90 adult wasps produced, and female proportions of 97.25% and 95.95%, by being reared on FAW eggs stored at 10 °C for three and five days, respectively. There were significant differences among storage/non-storage treatments in biological parameters (emergence rate: *F*_4,145_ = 8.119, *p* < 0.001; number of adults produced: *F*_4,145_ = 9.616, *p* < 0.001; female proportion: *F*_4,145_ = 5.450, *p* < 0.001). While there was no significant difference in biological parameters between the control and cold storage for 3 and 5 days, the emergence rate was *p* = 0.740 (3 d) and 0.168 (5 d); and the number of adults produced was *p* = 0.901 (3 d) and 0.969 (5 d); female proportion was *p* = 1.000 (3 d) and 0.983 (5 d). However, 11.75% of FAW eggs stored at 10 °C for three days hatched into larvae (Figure 4), reducing the number of available host eggs for rearing parasitoid wasps. Therefore, using FAW eggs stored at 10 °C for five days was the most suitable cold storage treatment of host eggs for rearing *T. remus*.

### 3.3. The Optimal Storage Conditions for T. remus Reared on Cold-Stored FAW Eggs

**Figure 5:**
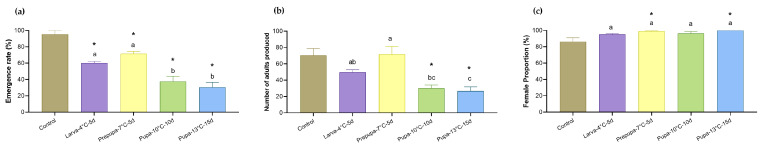
Reproductive parameters (**a**), emergence rate (**b**), number of adults produced, and (**c**) female proportion of *Telenomus remus* parasitizing *Spodoptera frugiperda* eggs under four cold storage conditions. Means (± SE) were calculated from three replicates. The asterisks indicate significant differences between storage treatments and control data based on Dunnett’s test at *p* < 0.05. Cold storage treatments with a different lowercase letter are significantly different using Tukey’s test at *p* < 0.05.

According to the comparison results of bioassays 1 and 2, the reproductive capacity of wasps in the four storage treatments (developmental stage/temperature/storage duration: larva-4 °C-5 d, larva-7 °C-5 d, pupa-10 °C-10 d, and pupa-13 °C-15 d) on non-stored FAW eggs showed no significant difference compared to the control. These conditions were used to conduct the cold storage experiment with *T. remus* parasitizing FAW eggs that had been stored at 10 °C for five days. Figure 5a shows that the emergence rates of *T. remus* in the four treatments were significantly affected by cold storage and the stored host eggs compared to the control (*p* = 0.001, *p* = 0.006, *p* < 0.001, and *p* < 0.001). With the extension of the storage period, the emergence rate decreased from 71.47% to 30.38%, even though the storage temperature was set to 13 °C. The emergence of adults in the treatment larva-7 °C-5 d and the number of adults produced were not affected by cold storage or the stored host eggs (*p* = 0.302, 1.000, 0.004, and 0.002) (Figure 5b). The proportions of females from the larva-7 °C-5 d and pupa-13 °C-15 d treatments were higher than those of the control (Figure 5c). No significant differences were observed between the other two treatments and the control (*p* = 0.225, 0.013, 0.061, and 0.002). Therefore, the optimal storage conditions for *T. remus* parasitizing stored FAW eggs were larvae stored at 7 °C for five days.

## 4. Discussion

Assessing the effects of cold storage on parasitic wasps and their hosts is one of the crucial components of quality control during mass reproduction [18,31]. In addition to investigating the impact of cold storage on different developmental stages of *T. remus* and the eggs of its host, the FAW, this study also focused on the impacts on the reproductive efficiency and shelf life of *T. remus* under the combined effects of cold storage and stored FAW eggs. When *T. remus* was stored with fresh FAW eggs as hosts, the pupae could be stored at 13 °C for 15 days without affecting reproductive efficiency. However, the emergence rate was only 30.38% when using the same storage treatment for *T. remus* parasitizing stored FAW eggs. These results indicated that the optimal cold storage conditions for *T. remus* on non-stored FAW eggs were unsuitable for *T. remus* on stored eggs. We suggest that the difference was caused by the use of cold-stored eggs to rear parasitic wasps. The results of bioassay 2 showed that, in addition to significantly reducing the hatching rate of the FAW, cold storage factors (temperature and storage duration) further affected the reproductive efficiency of *T. remus*. Using FAW eggs stored at 10 °C for only five days, the results for *T. remus* were similar in terms of the emergence rate, the number of adults produced, and the proportion of females to those using newly laid eggs. The maximum storage duration, including host and parasitoid wasps, was 10 days when *T. remus* parasitized stored FAW eggs. Compared with eggs from other hosts, FAW eggs have a shorter storage period. Chen et al. [27] reported that the eggs of *S. litura* could be stored at 11 °C for nine days and could be used to rear *T. remus*. The parasitism of *T. remus* on rice moth eggs stored at 10 °C for 21 days was the same or even higher than that of fresh eggs [25]. This demonstrated that cold storage affects the quality of the host and thus further affects the reproductive efficiency of the parasitoid. FAW eggs can only be cold-stored at 10 °C for five days before they are suitable for rearing parasitic wasps. The results were in accordance with the hypothesis that the duration of cold storage of host eggs may have an impact on the reproductive efficiency of parasitic wasps [32,33]. Therefore, cold storage of FAW eggs is not conducive to prolonging the shelf life of *T. remus* wasps. Compared with stored FAW eggs, it is better to rear *T. remus* wasps on fresh FAW eggs. Previous studies have generally focused on the impact of cold storage of host eggs on the reproductive efficiency of *T. remus* [23,24,25,27,34]. Our study is the first to analyze the comprehensive effects of cold storage of host eggs and cold storage factors on *T. remus*, and the results were consistent with our original hypotheses.

When parasitizing the eggs of *S. litura*, the optimum storage conditions for *T. remus* were adults for seven days at 8 °C or 11 °C, 1st instar larvae for 21 days at 14 °C [28], and pupae for 21 days at 14 °C [34]. In contrast, when *T. remus* employed the eggs of the rice moth as the host, the storage duration was the shortest [25]. However, when parasitizing *S. litura*, the storage duration was the longest, but with a higher storage temperature. The pupae of *T. remus* parasitizing rice moth eggs could be stored for seven days at 10 °C, while the adults could only be stored for less than four days at 5 °C or 10 °C [25]. When *T. remus* parasitizes FAW eggs, the cold storage temperature should not be lower than 10 °C, and the storage period should be less than 15 days. This may be related to the difference in cold tolerance of the host species.

Our results indicated that the response of *T. remus* to low temperatures varied according to the development stage and the duration of storage; as the temperature decreased, the shorter the time that *T. remus* could be stored. Pupae are more resistant to cold, while the larvae of *T. remus* are more sensitive to cold storage than other developmental stages. Therefore, short-term cold storage is recommended for *T. remus* due to its weak tolerance to cold. Parasitoid wasp species respond differently to temperature [15]. Other *Telenomus* species, such as pupal and adult *Telenomus busseolae* Gahan, could be stored for four weeks at 4 or 8 °C and up to 12 weeks at a temperature of 12 °C [35]. At 15 °C, *Telenomus podisi* (Ashmead) pupae could be stored for up to five months, but with the extension of storage time, the fecundity of the wasps decreased significantly (approx. 80%) [23]. These results reflect interspecific differences in cold tolerance.

We believe that simultaneously analyzing the hatching rate of the host and the reproductive efficiency of the parasitoid wasps can accurately determine the cold resistance of the host eggs and the storage conditions that are optimal for maintaining the freshness of host eggs used for rearing parasitoid wasps. The present results demonstrate that the storage conditions of 10 °C for five days could not only keep the FAW eggs fresh but also have the effect of ‘embryo killing’, which is conducive to the reproduction of parasitoid wasps [36]. Our previous study found that 12.85 °C was the developmental threshold temperature for FAW eggs (unpublished). Above this temperature, FAW eggs continue to develop, making them unsuitable for rearing parasitic wasps.

In the present study, which employed similar experimental setups to those of Lü et al. [26] and Chen et al. [28], we investigated the effects of storage factors and their interactions. We found that the proportion of females was unaffected by the host developmental stages for *T. remus* parasitizing freshly laid FAW eggs during cold storage, but this was affected by the three-way interaction of temperature, developmental stages, and storage duration. While it has been reported that the proportion of female *T. remus* reared on *S. litura* eggs was largely unaffected by cold storage [28], the prolongation of cold storage may affect the survival of the parasitoid and significantly reduce the survival rate of females [37]. Meanwhile, the proportion of females thus was affected by the storage duration and low temperature, whether *T. remus* parasitized freshly laid or stored eggs.

## 5. Conclusions

In summary, *T. remus* and its host, *S*. *frugiperda*, are intolerant to cold storage, and thus it is difficult to extend the shelf life via storage. The optimal storage conditions for *T. remus* wasps parasitizing non-stored FAW eggs comprised pupae stored at 13 °C for 15 days. It is feasible to keep FAW eggs fresh at 10 °C for five days, and these can be used to rear *T. remus* without affecting the emergence rate, the number of adults produced, or the proportion of females. However, the larvae of *T. remus* can only be stored at 7 °C for five days when reared on stored FAW eggs. Therefore, the maximal shelf life of *T. remus* parasitizing cold-stored FAW eggs is only 10 days.

The present study demonstrated that the reproductive capacity of the parasitoid was significantly affected by the dual stresses of cold storage and stored host eggs. Different storage conditions should be used when rearing *T. remus* on stored and non-stored eggs. We suggest rearing *T. remus* with freshly laid FAW eggs for short-term cold storage, as this can extend the shelf life by 15 days. This study offers new insights concerning the usage of *S*. *frugiperda* for mass rearing of its natural enemy, *T. remus*. The method can also be applied to other *Telenomus* species and to other parasitic wasps to accurately evaluate their reproductive capacity and shelf life during cold storage. Therefore, different cold storage strategies can be adopted to flexibly use parasitic wasps in biological control. Nevertheless, the application of the present results has several limitations. For example, due to the insufficient egg masses that met the experimental requirements, more replicates were not performed in bioassays 1 and 3, and the control potential of *T. remus* after cold storage was not investigated. Therefore, additional experiments need to be performed in the future, as well as laboratory and field validation of the control effectiveness of *T. remus*.

## Figures and Tables

**Table 1:** Factors and levels of storage treatments for *Telenomus remus*.

Level	Factors		
	Temperature (°C)	Developmental Stages (from the Day of Parasitism)	Storage Duration (d)
1	13 ± 1	Larva (2 d)	5
2	10 ± 1	Prepupa (4 d)	10
3	7 ± 1	Pupa (6 d)	15
4	4 ± 1	Late pupa (8 d)	20
5	27 ± 1 (control)	/	/

**Table 2:** Reproductive parameters of *Telenomus remus* parasitizing cold-stored *Spodoptera frugiperda* eggs.

Biological Parameters	Storage Temperature	Storage Duration			
		3 d	5 d	7 d	9 d
Emergence rate (%)	Control (27 °C)	84.21 ± 4.26			
	13 °C	66.06 ± 4.66 a *	41.54 ± 6.30 b *	57.97 ± 6.64 ab *	36.18 ± 5.54 b *
	10 °C	82.87 ± 2.79 a	76.05 ± 3.21 ab	61.32 ± 6.32 bc *	57.01 ± 6.74 c *
	7 °C	42.14 ± 4.62 a *	44.81 ± 5.73 a *	37.07 ± 6.96 b *	21.52 ± 6.26 b *
	4 °C	62.87 ± 5.51 a *	65.51 ± 6.35 a	32.01 ± 6.91 b *	23.31 ± 6.79 b *
Number of adults produced	Control (27 °C)	28.13 ± 3.29			
	13 °C	18.77 ± 2.69 a	11.77 ± 2.60 bc *	17.13 ± 2.89 ab *	7.30 ± 2.53 c *
	10 °C	26.30 ± 3.46 a	27.90 ± 3.37 a	19.50 ± 3.33 ab *	10.50 ± 2.02 b*
	7 °C	10.40 ± 1.78 a *	13.87 ± 3.04 a *	10.87 ± 2.50 ab *	3.33 ± 1.39 b *
	4 °C	12.87± 1.81 a *	12.20 ± 2.26 a *	2.37 ± 0.89 b *	1.27 ± 0.49 b *
Female proportion (%)	Control (27 °C)	96.65 ± 2.38			
	13 °C	90.89 ± 4.44 a	68.94 ± 8.39 a *	80.29 ± 6.79 a	73.13 ± 8.19 a *
	10 °C	97.25 ± 1.03 a	95.95 ± 1.33 a	77.21 ± 7.59 ab *	69.77 ± 8.11 b*
	7 °C	89.76 ± 5.13 a	83.10 ± 6.90 a	54.01 ± 8.93 b *	26.67 ± 8.21 b *
	4 °C	89.59 ± 2.03 a	86.67 ± 2.20 a	47.50 ± 9.00 b *	31.39 ± 8.34 b *

* Storage treatments vs. control data with an asterisk differed significantly according to Dunnett’s test at *p* < 0.05. Storage treatments with a different lowercase letter under the same temperature are significantly different using Tukey’s test at *p* < 0.05.

## Data Availability

The data presented in this study are available in this article.

## Supplementary Materials

The following supporting information can be downloaded at: https://www.mdpi.com/article/10.3390/insects15040233/s1, Table S1: Multivariate ANOVA of effects of three cold storage factors on reproductive parameters of *Telenomus remus* parasitizing fresh FAW eggs; Table S2: Multivariate ANOVA of effects of two cold storage factors on hatching rate of *Spodoptera frugiperda*; Table S3: Multivariate ANOVA of effects of two cold storage factors on reproductive parameters of *Telenomus remus* parasitizing stored FAW eggs.
