# Supplementary material for: Dual Effects of Cold Storage and Stored Host Eggs of Spodoptera frugiperda (Smith) (Lepidoptera: Noctuidae) on the Reproductive Capacity of Telenomus remus Nixon (Hymenoptera: Scelionidae)
Source: Insects. 2024 Mar 28;15(4):233. doi: 10.3390/insects15040233 (PMC11049916; doi:10.3390/insects15040233)
Supplement: Supplementary file 1 [file insects-15-00233-s001.zip › insects-2925623-supplementary.pdf]

Supplementary information

Ranran Qiu [1,2], Jun Li [1,*], Nicolas Desneux [3], Liansheng Zang [4], Xiaofang He [2] and Xin Lü [1,*]

[1] Guangdong Key Laboratory of Animal Conservation and Resource Utilization, Guangdong Public Laboratory of Wild Animal Conservation and Utilization, Institute of Zoology, Guangdong Academy of Sciences, 105 Xingang Road West, Guangzhou 510260, China
[2] College of Plant Protection, South China Agricultural University, Guangzhou 510642, China;
[3] Université Côte d'Azur, INRAE, CNRS, UMR ISA, 06000 Nice, France
[4] National Key Laboratory of Green Pesticide, Key Laboratory of Green Pesticide and Agricultural Bioengineering, Ministry of Education, Guizhou University, Guiyang 550025, China
* Correspondence: junl@giabr.gd.cn (J.L.); greenhopelv@163.com (X.L.)

Table S1. Multivariate ANOVA of effects of three cold storage factors on reproductive parameters of *Telenomus remus* parasitising fresh FAW eggs.

Biological parameters	Factors	df	*F*	*P*
Emergence rate (%)	A	3	48.47	<0.001
	B	3	335.71	<0.001
	C	3	101.53	<0.001
	A*B	9	13.62	<0.001
	A*C	9	1.76	<0.001
	B*C	9	13.88	<0.001
	A*B*C	27	4.04	<0.001
Number of adults produced	A	3	43.51	<0.001
	B	3	366.93	<0.001
	C	3	109.14	<0.001
	A*B	9	7.5	<0.001
	A*C	9	5.00	<0.001
	B*C	9	24.9	<0.001
	A*B*C	27	6.00	<0.001
Female proportion (%)	A	3	1.38	0.253
	B	3	81.78	<0.001
	C	3	16.76	<0.001
	A*B	9	2.36	0.017
	A*C	9	1.62	0.117
	B*C	9	16.06	<0.001
	A*B*C	27	3.62	<0.001

*A, developmental stage; B, storage duration; C, storage temperature

Table S2. Multivariate ANOVA of effects of two cold storage factors on hatching rate of *Spodoptera frugiperda*.

Biological parameters	Factors	df	*F*	*P*
FAW egg hatching rate (%)	A	3	21.70	<0.001
	B	3	32.24	<0.001
	A*B	9	4.22	<0.001

*A, storage duration; B, storage temperature

Table S3. Multivariate ANOVA of effects of two cold storage factors on reproductive parameters of *Telenomus remus* parasitizing stored FAW eggs.

Biological parameters	Factors	df	*F*	*P*
Emergence rate (%)	A	3	21.11	<0.001
	B	3	19.39	<0.001
	A*B	9	2.55	0.007
Number of adults produced	A	3	33.30	<0.001
	B	3	25.01	<0.001
	A*B	9	2.77	0.004
Female proportion (%)	A	3	28.16	<0.001
	B	3	8.47	<0.001
	A*B	9	3.96	0.017

*A, storage duration; B, storage temperature
